# Supplementary material for: Human Primary Astrocytes Differently Respond to Pro- and Anti-Inflammatory Stimuli
Source: Biomedicines. 2022 Jul 22;10(8):1769. doi: 10.3390/biomedicines10081769 (PMC9331936; doi:10.3390/biomedicines10081769)
Supplement: Supplementary file 1 [file biomedicines-10-01769-s001.zip › biomedicines-1762084-supplementary.pdf]

A)

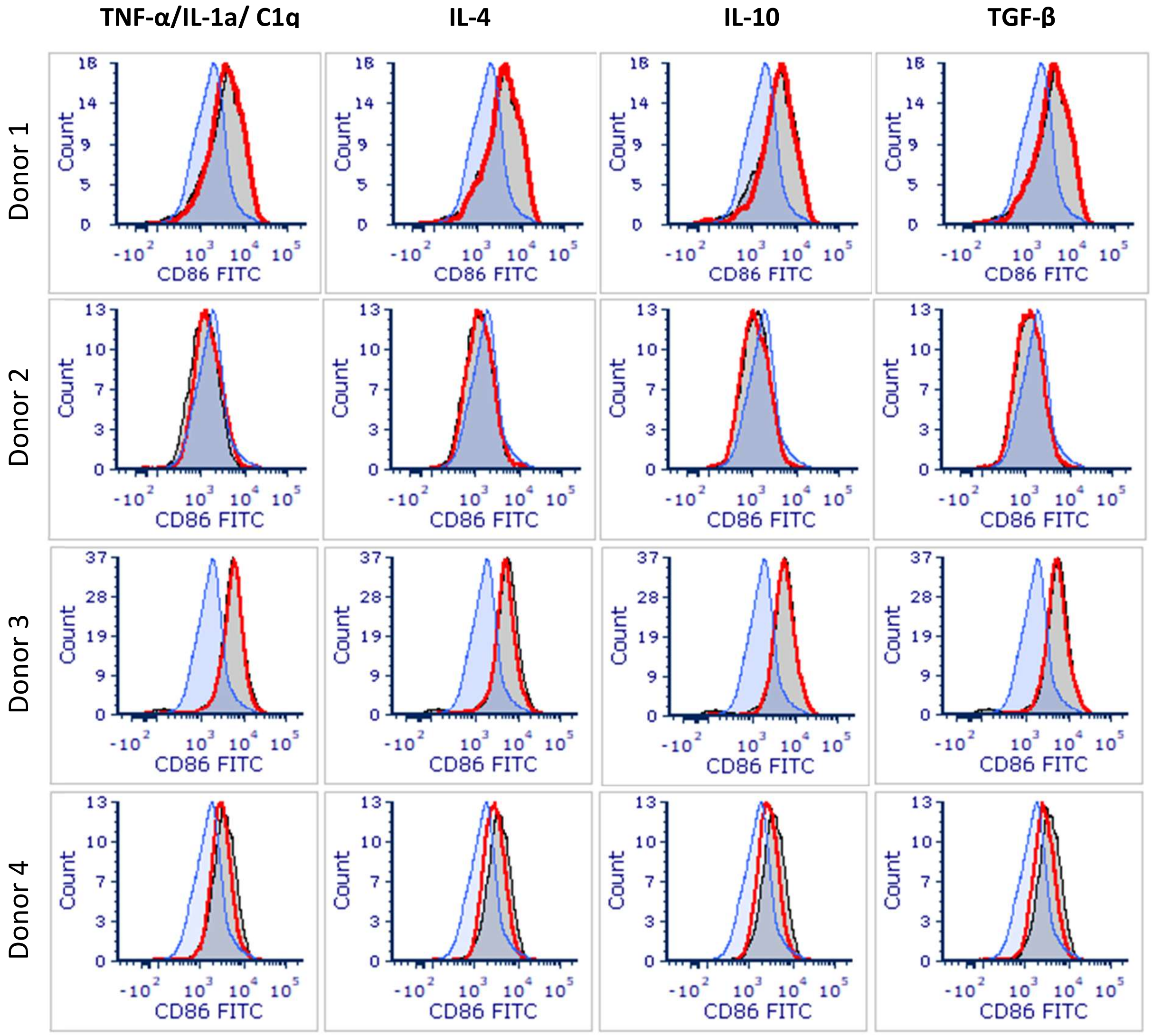
TNF-α/IL-1a/ C1q
IL-4
IL-10
TGF-β
Donor 1
Donor 2
Donor 3
Donor 4
Count
CD86 FITC
-10²
10³
10⁴
10⁵

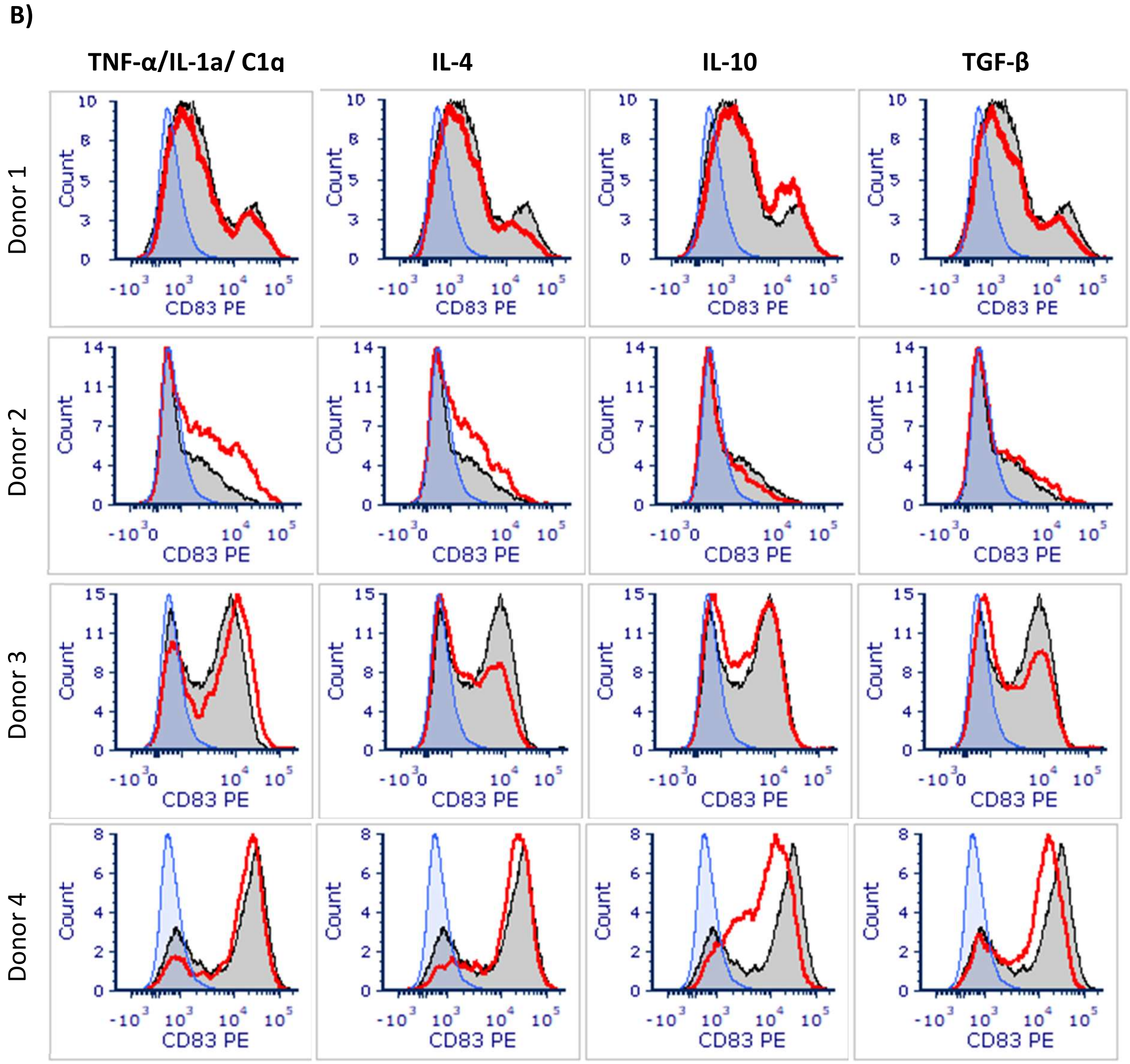
B)
TNF-α/IL-1a/ C1q
IL-4
IL-10
TGF-β
Donor 1
Donor 2
Donor 3
Donor 4
Count
CD83 PE

C)

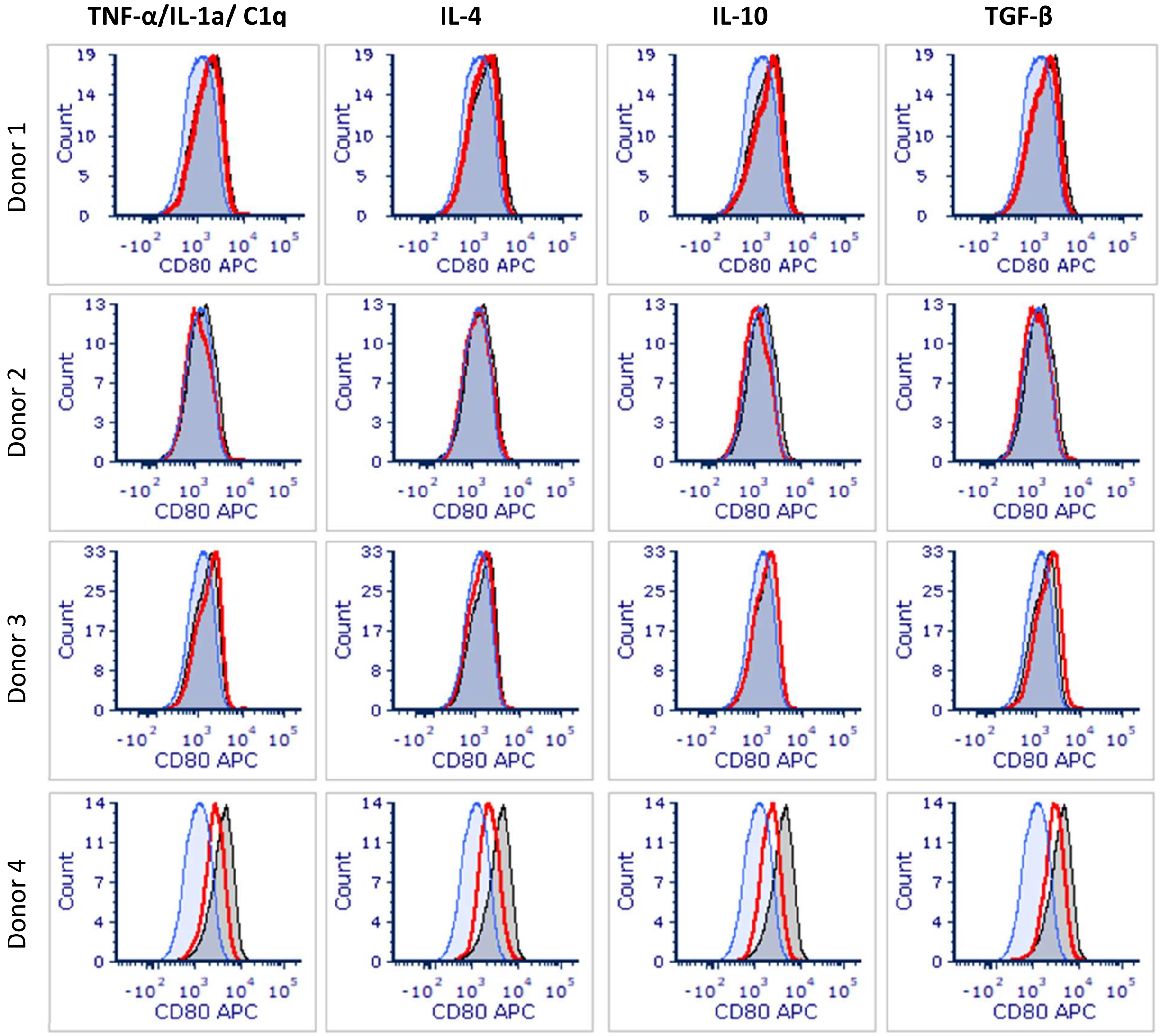

TNF-α/IL-1a/ C1q
IL-4
IL-10
TGF-β
Donor 1
Donor 2
Donor 3
Donor 4
Count
CD80 APC

D)

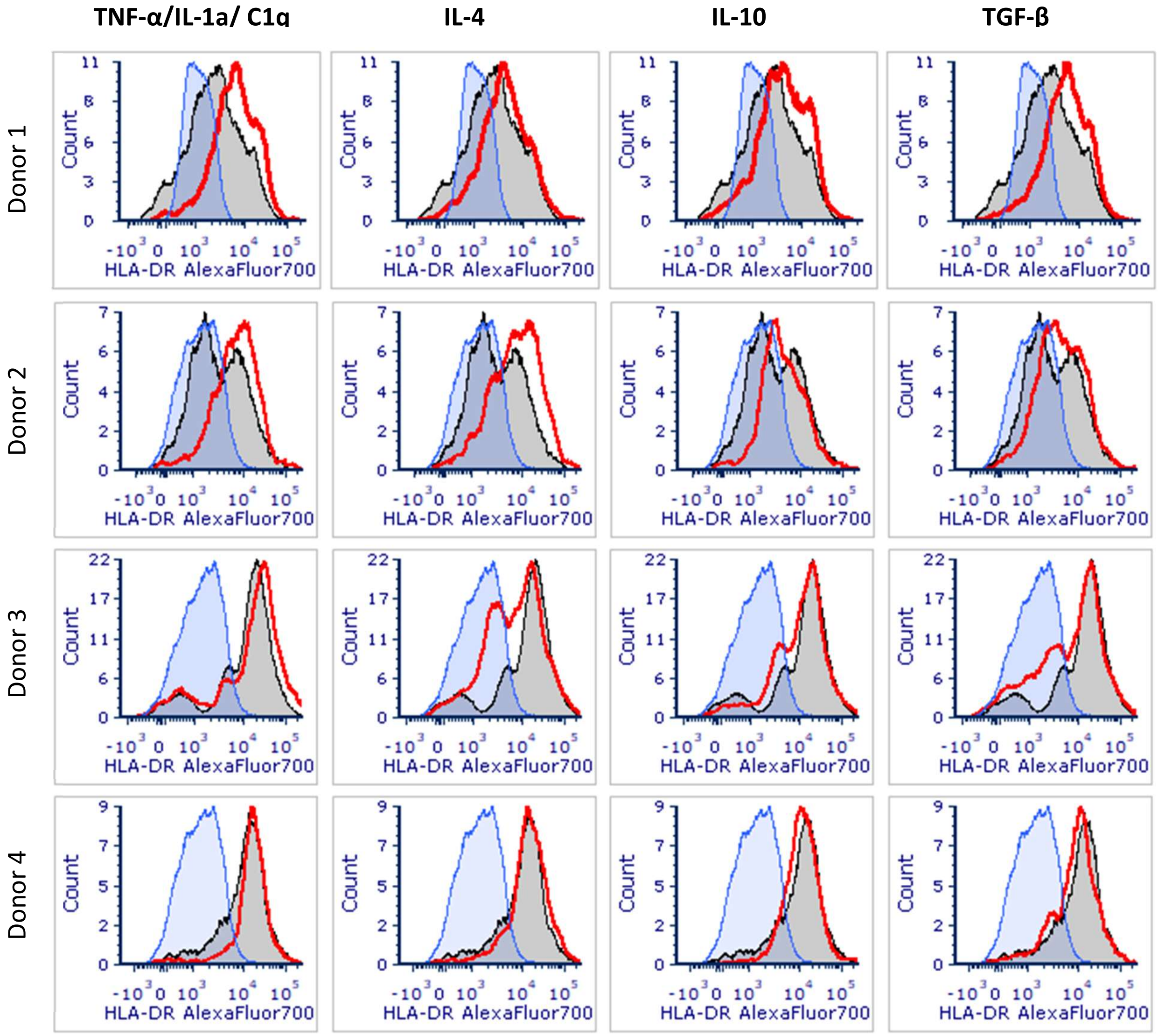

Supplementary Figure S1. Expression of surface co-stimulatory molecules: CD86 (A), CD83 (B), CD80 (C) and HLA-DR (D) on astrocyte cells in response to various cytokine environment. Representative histograms for 4 astrocyte's donors. Blue-filled histograms represent cells stained with isotype control antibodies, grey-filled histograms represent molecule expression on astrocytes grown in non-stimulatory conditions (culture medium), red-line histograms represent molecule expression on astrocytes grown in cytokine-enriched conditions.
